# The Plague at Bengazi

**Published:** 1858-11

**Authors:** 


*The Plague at Bengazi.*—We extract the following statements from the *Gazette Médicale d' Orient:* "There is unfortunately no doubt as to the kind of epidemic which has for some time been raging at Bengazi; it is certainly the plague. The Ottoman government received, on the 23d of July, the report of the commissioners who were sent to the spot; and this report is certainly explicit. The usual characters of the plague are thus described: High fever, wandering, prostration, vomiting, buboes, petechiæ, anthrax, the latter symptoms being less frequently met with than the former. The epidemic presents all the features of the plague, with its malignant character, its rapid course, and its spreading tendencies. The disease not only reigns at Bengazi, but has broken out in three of the four or five districts of which the province is composed. Derna, a seaport town of between ten thousand and fifteen thousand inhabitants, has been especially visited. Bengazi had formerly a population of twelve thousand souls; at the time of the arrival of the commissioners, the inhabitants numbered barely four thousand, owing to emigration and the numerous fatal cases. At least fifteen hundred persons had been attacked since the outbreak of the disease; and eight hundred of these had died. At Bengazi, the disease had broken out in the course of May, and had reached its highest point of intensity towards the 20th of June, when from twenty to thirty persons died per diem. From that period, the epidemic raged with undiminished violence; and towards the middle of July, there were only eight deaths per diem, out of a population reduced to about four thousand souls. But, while the disease decreased at Bengazi, it spread to the surrounding country, especially on account of the flight of a great many inhabitants of the town who scattered themselves in the neighboring districts. Such are the main facts which have transpired. Ample details will shortly be found in an elaborate pamphlet by the commissioners, which is said to be preparing."—*Medical News and Library.*

